# Survivorship of Patients After Long Intensive Care Stay With Exploration and Experience in a New Zealand Cohort (SPLIT ENZ): Protocol for a Mixed Methods Study

**DOI:** 10.2196/35936

**Published:** 2022-03-17

**Authors:** Lynsey Sutton, Elliot Bell, Susanna Every-Palmer, Mark Weatherall, Paul Skirrow

**Affiliations:** 1 Intensive Care Unit Level 3, Wellington Regional Hospital Capital and Coast District Health Board Wellington New Zealand; 2 Department of Psychological Medicine University of Otago Wellington New Zealand; 3 Department of Medicine University of Otago Wellington New Zealand

**Keywords:** COVID-19, critical illness, disability, intensive care unit, survivorship, Post Intensive Care Syndrome

## Abstract

**Background:**

*Post Intensive Care Syndrome* (PICS) was defined by the Society of Critical Care Medicine in 2012 with subsequent international research highlighting poor long-term outcomes; reduced quality of life; and impairments, for survivors of critical illness. To date, there has been no published research on the long-term outcomes of survivors of critical illness in New Zealand.

**Objective:**

The aim of this study is to explore long-term outcomes after critical illness in New Zealand. The primary objectives are to describe and quantify symptoms and disability, explore possible risk factors, and to identify unmet needs in survivors of critical illness.

**Methods:**

This will be a mixed methods study with 2 components. First, a prospective cohort study of approximately 100 participants with critical illness will be followed up at 1, 6, and 12 months after hospital discharge. The primary outcome will be disability assessed using the World Health Organization Disability Assessment Scale 2.0. Secondary outcomes will focus on mental health using the Hospital Anxiety and Depression Scale and the Impact of Events Scale-revised, cognitive function using the Montreal Cognitive Assessment (Montreal Cognitive Assessment–BLIND), and health-related quality of life using the European Quality of Life-Five Dimension-Five Level. The second element of the study will use qualitative grounded theory methods to explore participants experiences of recovery and highlight unmet needs.

**Results:**

This study was approved by the New Zealand Northern A Health and Disability Ethics Committee on August 16, 2021 (21/NTA/107), and has been registered with the Australian New Zealand Clinical Trials Registry on October 5, 2021. SPLIT ENZ is due to start recruitment in early 2022, aiming to enroll 125 patients over 2 years. Data collection is estimated to be completed by 2024-2025 and will be published once all data are available for reporting.

**Conclusions:**

Although international research has identified the prevalence of PICS and the extent of disability in survivors of critical illness, there is no published research in New Zealand. Research in this field is particularly pressing in the context of COVID-19, an illness that may include PICS in its sequelae.

**Trial Registration:**

Australian New Zealand Clinical Trials Registry ACTRN1262100133588; https://www.anzctr.org.au/Trial/Registration/TrialReview.aspx?id=382566&showOriginal=true&isReview=true

**International Registered Report Identifier (IRRID):**

PRR1-10.2196/35936

## Introduction

### Overview

In-hospital mortality for patients treated in intensive care units (ICUs) in New Zealand and Australia is low, with a high proportion of patients surviving to hospital discharge and beyond [1,2]. In part, due to population aging, patients are presenting to the ICU who are increasingly complex [3]. Those with advanced age, medical frailty, and multiple comorbidities are more likely to have high acuity, have long ICU stays, and importunate reliance on multi-organ support and mechanical ventilation [4,5]. The term *persistently critically ill* (PerCI) is used to describe patients who, in addition to a long length of stay in ICU, are at high risk for ongoing disability and poor quality of life after discharge from the hospital [6,7]. This complex group have a variety of initial conditions and are a growing proportion of patients treated in the ICU [2]. Although different cutoff points for length of stay have been used to define them [3,4], it is generally considered that around days 6-10 of the ICU stay represents the juncture at which the patient moves from being acutely ill to being PerCI [4]. Although they account for only 5% of total patients presenting with critical illness in Australia and New Zealand, they use 33% of all ICU bed days and 15% of all hospital bed days [3,4]. Approximately 1 in 6 patients meet the criteria for being PerCI in Australasian ICUs at any time [3,5,6].

The recovery and survivorship journey of these patients is challenging and burdensome. Even before the COVID-19 pandemic, there had been a growing emphasis on the high morbidity and poor quality of life for patients who have been critically ill [8]. A decade ago, Cuthbertson et al [9] reported that quality of life was significantly degraded in patients who are critically ill up to 5 years after critical illness. Herridge et al [10] reported that this was compounded by exercise limitation and physical and psychological sequelae and was associated with increased use of health care services. Other studies have also consistently reported similar poor outcomes across multiple areas of functioning, including mental health, neurocognitive, social, and physical domains [8,11-13]. Since an inaugural stakeholder meeting of critical care experts from the Society of Critical Care Medicine [14], this constellation of poor outcomes following ICU discharge was defined as the *Post Intensive Care Syndrome* (PICS).

After a successful run with a COVID-19 elimination strategy, the spread of the Delta variant prompted the New Zealand government to move to a minimization and protection approach. The impact of this on ICUs in New Zealand is uncertain; however, COVID-19 is likely to become endemic worldwide [15]. Evidence from overseas is that patients who are critically ill with COVID-19 exhibit PICS-like symptoms leading to impairment and disability once they are home [16]. The mechanism for these long-term impairments is still unclear, and debate about what may be long COVID-19 and what is PICS continues [17]. Irrespective of the cause of presentation to the ICU, nearly one-third of patients who are critically ill will go on to experience an element of PICS in their recovery, which warrants urgent attention [12].

### Background to the Study

Much of the understanding of PICS comes from follow-up clinics in the United Kingdom and the United States [18]. Published research consistently reports long-term impairments and other negative outcomes in patients with critical illness. Initial studies explored impairments related to specific symptoms such as fatigue, memory loss, cognitive dysfunction, depression, anxiety, posttraumatic stress disorder (PTSD), insomnia, prolonged ICU-acquired neuromuscular weakness, and alopecia [19-36]. However, more recently, a unifying definition has been used to better characterize domains of cognitive, mental health, and functional sequelae for PICS [14,37]. For example, in a multicenter cohort study, Marra et al [12] explored and quantified recovery outcomes in 406 survivors of critical illness. This study did not recruit patients with pre-existing impairments in baseline activities of daily living and cognitive dysfunction; hence, it was able to explore the role of critical illness in the development of new impairments. Three months after discharge, between 25% and 33% of patients had new cognitive impairment, new functional disabilities, or symptoms of depression, with many of these problems persisting after 12 months. Although most patients were assessed as having problems in 1 PICS domain (39% and 35% at 3 and 12 months, respectively), a substantial proportion of patients had problems in 2 domains (19% and 16% after 3 and 12 months, respectively), whereas a smaller group of patients had problems in all 3 domains (6% and 4% at 3 and 12 months, respectively).

Patient-centered outcomes in survivorship research are strongly emphasized [38]. Several studies have reported low quality of life, high levels of disability, and low rates of return to work in relation to PICS [8,9,22,30,39-42]. Hodgson et al [8] explored the concept of PICS and mapped patient difficulties to the World Health Organization International Classification of Functioning Disability and Health. Disability was assessed among 262 Australian survivors of critical illness after 6 months using the World Health Organization Disability Assessment Schedule (WHODAS) 2.0 [43]. The WHODAS was developed to measure disability across six major life domains: cognition, mobility, self-care, interpersonal relationships, work and household roles, and participation in society. The study reported disability was highly prevalent in survivors after six months, with 75% of participants experiencing variable levels of disability. More specifically, 50% experienced mild disability and 25% had moderate to severe disability. Those with moderate to severe disability were more likely to have a history of depression and anxiety and a longer duration of mechanical ventilation; a worse health-related quality of life; and significant reductions in mobility, personal care, and activities. Furthermore, only 40% of the patients had returned to work or study because of their disabilities, consistent with results reported by other studies [27,39-41,44,45]. Return to work has important implications not only for the individual but also for the family, wider community, and society at large.

### The Experience of Recovery

Qualitative work exploring patient accounts of recovery and PICS, compliments and improves the understanding of recovery after ICU treatment. This type of research adds depth and context to quantitative findings and allows patients to tell their own stories of recovery, coping, and transition to a new life. Several qualitative studies report that individual patient experiences can have elements that are unique and that the pace of recovery and trajectory are different from one person to the next [46]. Themes that are common to ICU survivorship include adjustment, acceptance, transition, and liminality (ie, giving up an old life) [47]. Kang and Jeong [48] described survivors’ recovery as being characterized by a need to embrace vulnerability, struggling through, moving from crisis to crisis, and progressing onward to a period of acceptance of their new selves. Through coping and internal and external support, survivors gained a new perspective on normality [49]. Only 1 qualitative study has been completed in New Zealand to date, which was solely focused on the ICU experience and interviews with the patient while still in the ICU [50].

Critical illness has a profound long-term impact on patients undergoing recovery. Not only do impairments related to cognition, mental health, and physical function create new and lasting disabilities but quality of life, return to work, and social functioning are also affected. To date, no studies of survivorship or long-term outcomes beyond mortality have been reported in New Zealand [51]. Research on the health system in New Zealand is important because of the specific nature of its population and health care system context. These unique aspects include the relatively low availability of ICU beds in relation to other health systems in high-income countries; the distinctive ethnic makeup within New Zealand society; and elements of the New Zealand health system, such as subsidized primary care services, a no-fault system for support after accidental injury, and the geographic distribution of the population. It is important that New Zealand–based qualitative and quantitative research explores the challenges that ICU patients in New Zealand face once home in recovering, both from and with PICS. Equally pressing is the need to understand the journey of New Zealand’s indigenous Māori population. To understand what Māori need to flourish during critical illness recovery, what support is needed and what are the unique health needs of our first nation population is urgently needed. This research protocol outlines and describes a mixed methods study to evaluate and quantify PICS in survivors of critical illness in New Zealand.

### Aims and Objectives

The aims of this study are as follows:

1. To estimate the proportion of intensive care survivors with moderate or severe disability at 1, 6, and 12 months after hospital discharge.
2. To describe the data distributions of relevant clinical variables and baseline characteristics of ICU survivors.
3. To understand the survivorship and recovery journey and describe unmet health needs in ICU survivors. A conceptual model of barriers to and facilitators of coping will be developed using grounded theory (qualitative study).

It is anticipated, and will be formally tested, that at least 20% of the adult patients who have had a prolonged stay in the ICU will experience moderate to severe disability in at least one domain of functioning in the year following critical illness.

### Trial Design

#### Quantitative Prospective Cohort Study

This study will be a mixed methods design with 2 components. The first component is a prospective cohort study using validated tools to assess and quantify the level of disability in the 12 months after critical illness. Outcomes will be assessed at three time points: 1, 6, and 12 months after hospital discharge. These times reflect clinically relevant points in the patient’s recovery, as identified in past research [31]. Disability will be quantified and assessed according to the WHODAS 2.0 (WHODAS-12). This will be the primary outcome measure.

Secondary outcomes will be health-related quality of life, mental health, and cognition assessed using validated tools at 1, 6, and 12 months. These tools have been chosen based on recommendations from expert committees and international research [37,52,53].

#### Qualitative Study

The second component is a qualitative study that will explore the process and experience of recovery for participants using the grounded theory [54]. The main purpose of the qualitative study is to identify the ongoing needs (met and unmet) in the year following critical illness, to facilitate the participant to tell their story of recovery, and to tease out the themes that drive recovery and the barriers that prohibit it. The grounded theory has been chosen as the methodology because it is well suited to social science research and has been used in several studies on ICU survivorship using both constructivist and classic approaches [47,48]. It is a rigorous and pragmatic model that incorporates a systematic but flexible approach. Moreover, the grounded theory is particularly well suited to research involving life transitions and the psychological responses to them [55] and has been used effectively alongside quantitative studies in mixed methods research [56]. This design will use a nested convenience sample conducting semistructured interviews between 6 and 8 months after discharge. Recruitment will involve sequential sampling initially, moving to theoretical sampling thereafter. Depending on thematic saturation, the grounded theory will dictate the number of participants required for the qualitative study.

This mixed methods study design has been chosen to highlight and quantify disability following a critical illness alongside qualitative data to bring context and provide a broad understanding of the recovery journey for survivors in New Zealand. Identifying the ongoing needs, barriers to recovery, and coping strategies will inform the development of resources or interventions to better support the long-term recovery of future patients with critical illness.

## Methods

### Participants, Interventions, and Outcomes

#### Study Setting

This will be a single-center prospective cohort study with all participants recruited at a 24-bed, tertiary ICU (Wellington ICU). This unit has a catchment population of approximately 1 million people in central New Zealand, admitting approximately 1800 people per year. Patients can be selected from within a geographic radius of 300 km, and research participants may be from across New Zealand’s lower North Island and upper South Island at follow-up. The study may be extended to other New Zealand ICU's if the impact of COVID-19 affects recruitment such that a sample size cannot be reached in Wellington.

#### Eligibility Criteria and Sample

The study sample will include all adult patients admitted to the Wellington ICU who meet the inclusion criteria within the recruitment period. This is likely to comprise a mix of patients with various conditions and acuity. Where possible, the inclusion and exclusion criteria have been designed to capture patients who are likely to have evidence of PICS while minimizing and excluding the effects of any pre-existing conditions that may worsen because of critical illness.

#### Inclusion Criteria

All participants will be adult (aged >18 years) ICU patients admitted to the Wellington ICU who have been in an ICU for 7 or more consecutive days or patients who were mechanically ventilated for >72 hours. This inclusion criterion has been designed to capture patients who are most likely to experience poor outcomes once they are home, based on previous research [57]. Patients who have been in another New Zealand ICU or critical care unit before retrieval to the Wellington ICU will be included if both admissions combined is ≥7 days.

Mechanical ventilation is defined as a positive pressure ventilation (PPV) mode via an endotracheal, nasotracheal, or tracheostomy tube. Patients who have been extubated from PPV for a period and then reintubated will also be included if both periods of PPV exceed 72 hours.

Patients with known depression or anxiety will be included because these low prevalence mental health conditions are common, and it would be unreasonable to exclude them. This is a limitation acknowledged in the study by Marra et al [12]. If the participant has experienced previous mental health issues but where mental health information may not be evident from their medical records, this will be ascertained at the first follow-up and the patient will be directly asked the following questions:

- Have you ever been diagnosed with a mental health problem by a physician or psychologist?
- If so, what was the diagnosis they made?

#### Exclusion Criteria

People are not eligible for the study if they present with the exclusion criteria outlined in Textbox 1.

**Exclusion criteria.**

**Exclusion criteria**

- <18 years
- Non-English speakers
- Not expected to survive their hospital stay as identified by an intensive care unit (ICU) senior medical officer once the inclusion criteria are met
- Have significant challenges for follow-up, for example, are prisoners or are homeless
- Have the following pre-existing conditions:
  Neuromuscular disorders, for example, muscular dystrophy, multiple sclerosis, myasthenia gravis, or Guillain Barré syndrome
  Neurodegenerative disease
  Psychiatric disease or intellectual disability in which patients are already mentally, cognitively, or functionally impaired before ICU admission (identified from the patient’s health history)
  Moderate to severe cognitive impairment, as recorded in the patient electronic health records and medical notes
- Presented to ICU with stroke, neurotrauma, status epilepticus, hypoxic or ischemic brain injury, or encephalopathy
- Have any other disease or disorder which, in the opinion of the principal investigator may influence the result of the trial

#### Intervention Description

This study has no active intervention but is based on collecting clinical data from eHealth sources and more in-depth responses to the research instruments that are described in the following sections. After informed consent is obtained, baseline data will be collected, including admission details, demographics, ethnicity, and baseline function (Charlson Comorbidity Index and Clinical Frailty Score). Clinical data collected will include diagnosis and the duration of therapies such as intubation, sedation, renal replacement therapy, antibiotic therapy, and mechanical ventilation. Data regarding time to negative polymerase chain reaction test (COVID-19 patients only) and complications during the ICU stay (delirium, ventilator-associated pneumonia, sepsis, and others) will also be collected.

Follow-up will be scheduled at 3 time points during the study. These are at 1, 6, and 12 months after discharge from the hospital. At these follow-up periods, the following tools or outcome measures will be completed by telephone: World Health Organisation Disability Assessment Scale (WHODAS) 2.0, Montreal Cognitive Assessment–BLIND, Hospital Anxiety and Depression Scale, Impact of Events Scale-revised, and European Research Foundation-Five Dimension-Five Level.

#### Qualitative Component

Participants will be approached at their 6-month follow-up, and after questionnaire completion, they will be invited to be part of a nested sample of the main study. Sequential sampling will be used initially, moving to the theoretical sampling approach, as per the grounded theory [54]. Thematic saturation will dictate the number of participants required for the qualitative study. All interviews will be undertaken by the principal investigator (PI) to ensure consistency. Participants will take part in audio recorded interviews using either face-to-face, Zoom, or telephone interviews, a method that is feasible or preferred for the participant and in line with any government-mandated COVID-19 restrictions.

#### Criteria for Discontinuing or Modifying Allocated Interventions

Participants will be contacted 1-2 weeks before the time of follow-up. Those who do not respond after 3 phone calls will be considered lost to follow-up and no further data will be sought. Data collected until that point will continue to be used.

Participants who have been readmitted to the hospital at the time of follow-up will continue to be enrolled in the study, with assessments deferred until they are discharged home again. If the participant has died, the date and causes of death will be recorded and data collected up to that point will be used, and the person will be withdrawn from the study.

As part of the informed consent process, participants will be told that they can withdraw at any time and do not need to provide any reason for withdrawal. Participants who wish to withdraw, as communicated verbally or in writing, will be withdrawn immediately. Data collected until that point will still be used and communicated to participants via the information or consent form.

#### Strategies to Improve Adherence to Interventions

The study has been designed to minimize the burden on participants. First, several methods of obtaining consent have been created so that participants can engage the way they find easiest and most convenient, for example, web-based, paper mail out, mobile phone SMS text messages, or other means. Second, the outcome measures and questionnaires have been chosen based on participant ease, tolerability, and feasibility in mind, while also ensuring that they are responsive and valid. Third, reminder texts and emails will be sent before all follow-ups. Fourth, participants will be able to select their preferred data collection method. If in-person interviews occur, the PI will travel to the participant (there is funding for travel granted in this study). As an additional incentive, a small gift will be offered to the participants in the qualitative study.

#### Relevant Concomitant Care Permitted or Prohibited During the Study

All relevant concomitant care and interventions will occur during the study in relation to the routine and expected clinical care of the participants, and participants will be advised to continue recovery and rehabilitation as advised by their care provider.

### Outcomes Measures for the Quantitative Study

There is currently no standardized, comprehensive tool to measure PICS, with >250 separate tools in existence [58]. Although research into validated tools such as PICS questionnaires is beginning to emerge, this research is limited and not generalizable to the New Zealand population [49]. The outcome measures chosen to emphasize a set of core outcome measures that are valid, responsive, and specific. This will ensure the same reproducible outcome measures, with comparability between studies and the generation of good quality meta-analyses [59].

Several international critical care expert committees have published recommendations in the core outcome sets (COSs) that should be used in research evaluating long-term outcomes in ICU survivors [37,52,53]. In addition, the development of COS for COVID-19 research is also emerging, with an Australian study evaluating 6-month outcomes after COVID-19 using WHODAS, European Research Foundation-Five Dimension-Five Level, Montreal Cognitive Assessment–BLIND, Hospital Anxiety and Depression Scale, and the Impact of Events Scale-6 with success [16]. WHODAS 2.0 and the secondary outcome measures used in both studies by Hodgson et al [8,16] will also be used for SPLIT ENZ, which have been shown to be reliable, valid, and responsive. Additional measures will include mortality and return to work (or study) (as assessed using the WHODAS 2.0). With guidance from the COS and other high-quality published research [8,12,16,37,52,53], the outcome measures chosen for this prospective cohort study are summarized in Table 1.

**Table 1:** Primary and secondary outcome measures.

Tool		Domain measured	Use and scoring
**Primary outcome measure**			
	WHODAS^a^ 2.0 [43]	Function	The 12-item WHODAS 2.0 covers 6 domains of functioning with scores from 0 (no difficulty) to 4 (extreme difficulty). The total score between 0 and 48, is then divided by 48 and multiplied by 100 to convert it to a percentage of maximum disability as follows: no disability (0%-4%), mild disability (5%-24%), moderate disability (25%-49%), severe disability (50%-95%), and complete disability 96% to 100%.
**Secondary outcome measures**			
	EQ-5D-5L^b^ [60]	Health-related quality of life	Measured in five domains: mobility, personal care, usual activities, pain, anxiety, and depression. Each dimension has 5 levels (*no problems*=1 to *extreme problems*=5). The EQ-5D-5L consists of two pages: the EQ-5D descriptive system and the EQ VAS^c^. The EQ VAS is used as a measure of overall self-rated health status as a numerical score.
	HADS^d^ [61]	Depression and anxiety	The HADS contains fourteen questions: 7 to assess anxiety and 7 for depression. For the 14 questions, a 4-point Likert scale (range 0-3) gives a possible score of 0 (none) to 21 (severe) for each of the two subscales: 0-7 indicate normal or no anxiety or depression symptoms, ≥8 to 10 indicate clinically significant anxiety or depression symptoms (borderline cases), and ≥11 indicate severe psychological distress.
	IES-r^e^ [62]	PTSD^f^	There are 22 questions that cover the three diagnostic clusters: intrusion; avoidance (8 questions each); and hyperarousal (6 questions). Respondents report on a 5-point Likert scale: *not at all* (item score 0) to *extremely* (4) how distressed they have been in the past 7 d in relation to a specific event. The IES-r yields a total score (ranging from 0 to 88) and subscale scores can also be calculated for the intrusion, avoidance, and hyperarousal subscales. The total mean IES-r score is the sum of the means of the 3 subscale scores. The maximum mean score on each of the 3 subscales is *4*, therefore the maximum *total mean* IES-r score is 12. A total IES-r score of 33 or more from a theoretical maximum of 88 signifies the likely presence of PTSD.
	MOCA^g^-BLIND [63]	Cognitive function	The total possible score is 22 points; a score of 18 or above is considered normal. Cutoffs have not been validated in patients who are critically ill.

^a^WHODAS: World Health Organization Disability Assessment Schedule.

^b^EQ-5D-5L: European Research Foundation-Five Dimension-Five Level.

^c^EQ VAS: EuroQol visual analogue scale.

^d^HADS: Hospital Anxiety and Depression Scale.

^e^IES-r: Impact of Events Scale-revised.

^f^PTSD: posttraumatic stress disorder.

^g^MOCA: Montreal Cognitive Assessment.

### Participant Timeline

**Figure 1:**
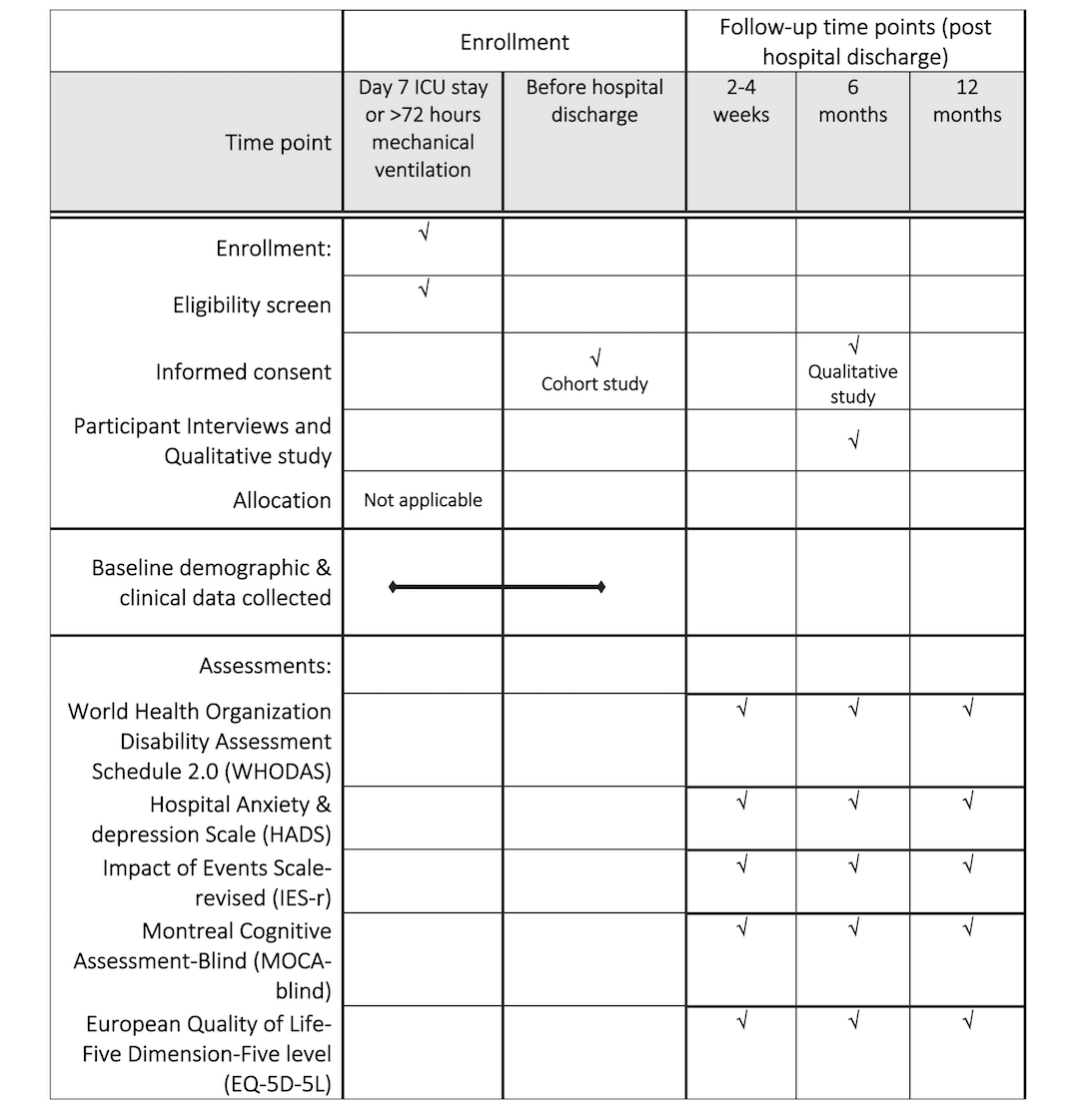
Timeline of SPLIT ENZ study schedule. ICU: intensive care unit.

The timeline of SPLIT ENZ study schedule is shown in Figure 1.

### Sample Size—Quantitative Study

All adult patients admitted to the Wellington ICU between February 2022 and February 2024 who are eligible will be invited to participate. Admission modeling suggests that this will give approximately 100 potential participants. The anticipated sample size of 100 gives a 95% CI for a proportion of plus or minus 10%. This sample size has 80% power to test that the proportion with PICS in less than approximately 10% or more than approximately 30% based on an assumed proportion of 20%.

### Recruitment

#### Overview

Despite the broad inclusion criteria, it is likely that recruitment may be slow and that completion to follow-up may also be low. Slow recruitment may reflect the patient cohort, which is typically the emphasis on studies of PICS and likely so with this study. These patients, historically termed PerCI [3], have the longest stays in the ICU but reflect only 5% of all patient admissions to the ICUs in Australia and New Zealand. In addition, New Zealand has the lowest proportion of ICU beds per 100 compared with the rest of the Organisation for Economic Co-operation and Development countries [64], which affects overall admission rates and potential recruitment of participants.

It is anticipated that a significant proportion of patients may also fail to reach study completion because of in-hospital death (but after ICU discharge) and death at home. International studies report variable mortality rates in the post-ICU year, between 20% and 50% for a variety of critically ill subpopulations [13,57,65]. One subpopulation in particular, patients with COVID-19 are also reported to have mortality rates as high as 30%, 6 months after discharge, and ICU mortality rates between 50% and 97% [66]. During the course of the study, the application of new therapies may change the survival experience of these patients with an uncertain effect on recruitment and follow-up [67]. The SPLIT ENZ team acknowledges these limitations and aims to ensure as many participants complete follow-up, by extending the recruitment period for up to 2 years. This should ensure that sufficient participants have data available at 6 months.

#### Qualitative Study Sample Size

Participants will be approached to participate in the qualitative study at the scheduled 6-month follow-up for the quantitative study. This sample will be generated sequentially from the main quantitative cohort (nested sample) and will be consented separately. Participants will be approached at the 6-month telephone follow-up, and verbal and written consent will be sought. Recruitment will continue, and interviews are completed until (thematic) data saturation is reached. As the grounded theory moves through the process of continuous recruitment, data collection, analysis, coding, and thematic exploration simultaneously, these processes inform the number of participants required to reach saturation of themes [54]. In this instance, the sample size is not preset, but it is likely to include an estimated sample of 15-20 participants.

#### Significance to Māori

Between 10% and 20% of patients admitted to the Wellington ICU each year are of Māori ethnicity. In New Zealand, Māori account for 17% of the total population [68] and experience health disparities across most major health, education, and psychosocial sectors. High rates of inequity, socioeconomic disparity, and increased barriers to access at all levels of health care exist for Māori [68]. Therefore, it is important that this study sample is reflective and inclusive to ensure that the voice of Māori participants is heard, something that is currently missing in New Zealand. The qualitative aspect of this study will be an important method by which we may understand the recovery journey, understand what helps Māori to flourish, and identify what unmet needs and barriers to recovery remain. Boosted sampling will be used to ensure that a high number of Māori participants are included in the qualitative part of the study sample. The researchers consulted with the Wellington, New Zealand Research Advisory Group for Māori, the Kaupapa Māori research network, and the Ngāi Tahu Research Consultation Committee at Otago University. This research protocol has been supported by these groups.

### Data Collection, Management, and Analysis

#### Data Collection Completed Once Deemed Eligible and Once the Patient has Consented

The following data will be extracted from the ICU database, electronic and paper-based patient notes, and ICU observation charts (Textbox 2).

**Data to be collected once eligible and after recruitment.**

**Initial details to be collected once patient meets eligibility criteria**

- Key intensive care unit (ICU) and hospital admission and discharge dates
- Contact details for patient and next of kin
- Demographics
- Diagnosis

**Subsequent data collection once patient consented**

- Clinical frailty score
- Apache II and Apache III scores
- Charlson Comorbidity Index and types of comorbidities
- Admission sequential organ failure score

**Length of stay and funding**

- Accident Compensation Corporation funding (yes or no)
- Hospital length of stay
- ICU length of stay

**Measures of clinical status or acuity**

- Number and type of clinical complications in the ICU (described and listed)
- PaO_2_/FiO_2_ ratio per day while mechanically ventilated
- Number of reintubations/failed extubation or extubations during ICU stay

**Duration (hours/days) of interventions**

- Renal replacement therapy (continuous or intermittent dialysis), extracorporeal membrane oxygenation, vasopressors, invasive hemodynamic monitoring, and antibiotic therapy duration
- Time to negative polymerase chain reaction test and if isolated for COVID-19
- Daily oxygen therapy, specifying high-flow nasal prongs and low-flow oxygen (ie, via nasal prongs), noninvasive ventilation and direct tracheostomy interface
- Oxygen free days
- Mechanical ventilation duration hours/days and mode
- Time to extubation and tracheostomy decannulation
- Tracheostomy weaning duration
- Sedation types and doses per day
- Mean Richmond Agitation Sedation scores per day
- Paralysis agents (neuromuscular blocking agents) doses and type per day
- Delirium duration hours/days as evidenced by the Confusion Assessment Method for ICU scores

**Other**

- Best daily ICU mobility score (if recorded)
- Chelsea Critical Care Physical Assessment Score (if completed at ICU discharge)
- If a patient diary was assigned to the patient in the ICU

#### Data to Be Collected at Follow-up

Follow-up data will consist of questionnaires administered by the PI over the telephone, using the primary and secondary outcome measures described in Table 1. The data will be recorded on data collection forms in accordance with the tool creators’ specifications after any required training has occurred. In addition, the participants will also be asked if they have tested positive for COVID-19, as this may influence the results of questionnaires.

#### Qualitative Study Data

The following key themes are explored based on previous research highlighting themes common to the recovery experience [47,48]:

- Experience of recovery overall
- Coping strategies and things that facilitate recovery (eg, spiritual, family, and social connectivity)
- Barriers, including met or unmet needs

Grounded theory will be undertaken to guide data collection during semistructured recorded interviews. Several data collection strategies will be used during the process: simultaneous collection and analysis of data, open and axial coding with comparative analysis (within cases and across cases), refining theoretical ideas, and memo writing [54,69]. Interviews will be audio recorded transcribed verbatim, coded, and checked for accuracy with another researcher. The PI will independently read and code the transcripts, the codes will be examined, and by an iterative process, the codes will be condensed into similar themes [69]. A second researcher will check the transcripts for truth and completeness. To achieve saturation of the themes, researchers will move back and forth between data collection and analysis, reidentifying themes and subthemes [69]. Work completed early in the study will inform subsequent recruitment using theoretical sampling data collection and analysis.

#### Outcome Measures for Qualitative Study

The following outcomes or themes were used to guide the interviews: ultimately, as part of the qualitative study, we will present a conceptual model of barriers and facilitators to coping for ICU survivors.

#### Plans to Promote Participant Retention and Complete Follow-up

Strategies will be used to maximize continued recruitment throughout the study. These include ensuring that consent is easy, follow-up is not arduous, and patient or family preferences are understood. Regular contact will also be made with participants to ensure engagement throughout the study period (text or mail reminders), and clear written information will be delivered before follow-up.

### Data Management

Patients will be allocated a unique study number by the PI. Study data and enrollment logs will be kept separate in a locked research office at the study center. Once the unique participant number is allocated, documentation will be deidentified and referred to only using that number. Data will be coded and entered into Microsoft Excel spreadsheets and Microsoft Word documents. Data will be stored securely in a locked research office for 10 years or in a password-protected secure computer file.

## Results

### Statistical Methods for Primary and Secondary Outcomes

#### Overview

Continuous variables will be described by mean and SD, median and IQR, and minimum to maximum values. Appropriate frequency histograms and boxplots will also be used to summarize the data distributions. Categorical variables will be described by numerators and denominators, and proportions will be expressed as percentages.

Proportions will be estimated together with CIs using standard binomial methods. It is anticipated that asymptotic methods for the CIs will be satisfactory; however, if there are many small frequency counts, exact binomial methods will also be used (aim I).

Associations between disability measured by the WHODAS and potential univariate predictors will be examined by logistic regression with disability categorized and moderate or severe versus lesser degrees of disability. As a sensitivity analysis, WHODAS will be treated on a continuous scale and ordinary regression used. For the latter, normality assumptions will be assessed by residual analyses to determine if a data transformation will be needed or if another form of regression such as ordinal regression might be more suitable. With an anticipated 25-30 participants with moderate or severe disability, this gives limited scope for multivariate analysis, but as discussed in the following sections, a more limited number of potential predictors will be used in a multivariate model to determine if associations remain after adjusting for confounding.

We selected a priori the following covariates as potential predictors of disability after intensive care admission: age, gender, ethnicity, APACHE II and III score, sequential organ failure score, duration of sepsis (days), frailty score, Charlson Comorbidity Index, length of ICU stay reported in days, duration of mechanical ventilation (in hours), duration of delirium (reported as days the patient was Confusion Assessment Method for the ICU positive), total doses of sedation, use of benzodiazepines, use of neuromuscular blocking agents, previous history of depression, anxiety, or PTSD (obtained from medical records, ICU database admission, and Medical Admissions Portal) and whether the ICU admission was for cardiothoracic surgery whereby the patient underwent cardiopulmonary bypass. Drug doses (sedative agents, benzodiazepines, and neuromuscular blocking agents) will be transformed into mean doses per day and analyzed over the number of days received.

Each of these potential predictors will be examined using univariate predictors with accompanying illustrative plots. In general, the analysis strategy will treat disability as a dichotomous variable and use logistic regression to estimate odds ratios for association and as discussed to explore linear regression and ordinal regression treating the WHODAS as a continuous response variable and possibly as an ordinal response variable. Although the primary interest is in disability after 12 months, the associations at earlier points, 1 month and 6 months, will also be estimated. At least one study has categorized disability based on the WHODAS-12 as none, mild, and moderate to severe disability [70]; however, it is likely to be more useful to explore if the instrument can be used on its native scale or use ordinal regression based on the full range of scores rather than other cutoff values.

Although not directly related to the study aims, mortality will also be assessed using the Kaplan–Meier curves and associated estimates of median or, where relevant, other percentiles, survival.

#### Subgroup Analyses

Subgroups of patients of interest will be those with a history of depression, anxiety or PTSD; those who have undergone postcardiac surgery and cardiopulmonary bypass in the preceding 3 months before ICU admission (and are at high risk for postoperative cognitive dysfunction) [71], those categorized by COVID-19 and non–COVID-19 illness, and ethnicity. Whether proportions with PICS or other outcomes differ in relation to these subgroups will be explored using interaction terms in the regression analyses.

#### Data Monitoring

Data monitoring for quality will be maintained within the SPLIT ENZ team, and there is no formal outside data monitoring committee that will be used as part of this study. No formal interim analysis will be performed as part of this study.

#### Reporting Adverse Events and Patient Safety

Any adverse events as part of this study will be discussed and managed within the SPLIT ENZ team and documented in the study file notes or data collection sheet. If there is a patient safety issue, this will be managed by the coordinating investigator in conjunction with the SPLIT ENZ team, and the escalation plan will be followed and documented.

There are no provisions for posttrial care, as this is a low-risk study. However, it is anticipated there may be a proportion of patients who require ongoing support from their health care provider (general practitioner [GP]) during the follow-up period. If the patient is found to be in distress with unmanageable symptoms at any of the follow-up periods, consent will be sought to contact the patient’s GP will be asked to provide help and treatment where possible. The PI will be responsible for ensuring that the GP is contacted and made aware, and a note to file will be created in the patient’s notes. At all study follow-up calls, the participant will be encouraged to have a support person with them if they wish.

### Ethics, Consent, and Dissemination

#### Research Ethics Approval and Protocol Amendments

This study has received full ethics approval from the New Zealand Northern A Health and Disability Ethics Committee on August 16, 2021 (21/NTA/107), and has been registered with the Australian New Zealand Clinical Trials Registry on October 5, 2021 (12621001335886). In light of the sudden outbreak of the Delta and Omicron strain of COVID-19 in New Zealand in August 2021, further protocol amendments were sought to ensure the overall study design, consent processes, and outcome measures were appropriate for patients admitted to the ICU with both COVID-19 and non–COVID-19 disease. Approval was granted for the protocol amendments on November 10, 2021, from the New Zealand Northern A Health and Disability Ethics Committee.

Any further changes to the protocol that may affect the conduct of the study or patient safety, including modifications to study objectives, study design, patient population, sample sizes, or study procedures, will require formal amendments to the protocol. Any such amendments will be agreed to by the SPLIT ENZ research group and must be approved by the Health and Disability Ethics Committee before implementation and notified to the health authorities and study sites in accordance with local regulations. The Australian New Zealand Clinical Trials Registry will also be updated with any relevant changes or updates.

Minor changes to the protocol (eg, corrections and clarifications that have no effect on the way the study is conducted) will be agreed to by the research group and logged on the Health and Disability Ethics Committee website.

#### Consent

During the recruitment period, the ICU patient database will be screened daily by the PI. Once potential participants have been identified as meeting the inclusion criteria, they will be screened to ensure that none of the exclusion criteria apply. If the person is eligible for recruitment, they and their families will be approached by the PI. It is highly likely that patients will be unable to provide consent at that time, so their family will be given information about the study and informed that once the patient is able to give consent, they will be approached by the PI. All consent processes will be managed by the PI.

An approach consistent with section 7.4 of the New Zealand Health and Disability Code [72] will be used to obtain consent. However, it is acknowledged that the consent process may require contingencies with the uncertainty around national COVID-19 restrictions and future lockdowns. There are 2 processes and contingencies to obtain consent in these circumstances. The following section outlines the different scenarios and their contingencies.

#### When Consent Can Be Gained in Hospital

Consent will be gained from prospective participants before discharge from the hospital (while recovering on a ward) when they are competent to give informed consent (ie, they are able to comprehend and communicate their wishes). The patient and family will be given all the necessary information about the study by the PI in a face-to-face meeting, and consent forms will be provided to the patient. The patient will be given sufficient time to think about participation, and if they are willing to take part, signed consent forms will be collected.

#### Where Consent Cannot Be Gained in Hospital

If the patient cannot consent while in Wellington hospital or they are transferred to another facility or domicile before the opportunity to gain written consent occurs, there are several options that will be used. First, an initial phone call to engage with the patient and the family will be made. If verbal consent is given, the consent form can be mailed out or emailed to participants with written information about the study. Prepaid envelopes will be provided for mailing back to the coordinating center. If the patient finds it easier to take a photo of the signed page and send it this way, it can be done via email or texting back to the PI. For participants who prefer an electronic method, a web-based consent process via the REDCap (Research Electronic Data Capture; Vanderbilt University) system has also been created.

#### Qualitative Study Consent

At the 6-month follow-up, the participant will be invited to further partake in qualitative interviews with the PI. All information will be mailed or emailed to the potential participants if they indicated that they would like to take part.

#### Confidentiality

All personal, demographic, and identifying data will be managed as stated under the *Data Management* section. Consent will be sought at the time of follow-up to alert the participant’s family physician if the researchers become aware of urgent or follow-up care that is needed, and this discussion will remain confidential. All reporting and publication of data will contain fully deidentified data and will undergo stringent peer review before publication in accordance with the journal’s editorial policy.

#### Access to Data

Patients will be allocated a unique study number by the PI. Study data and enrolment logs will be kept separate in a locked research office at the study center. Once the unique participant number is allocated, documentation will be deidentified from there on in and referred to using that number. The data will be archived for 10 years in accordance with the University of Otago guidelines. Only the PI will have access to the raw data, but the wider SPLIT ENZ team will have access to the final data set that will be published once the study concludes. Outside investigators may contact the PI for any data at the conclusion of the study and the publication of the results.

#### Composition of the Coordinating Center

The coordinating center consists of the PI supported by the SPLIT ENZ supervisory team (PS, SEP, EB, and MW). Although there is no formal outside trial steering committee for this study, all trial design queries and quality control oversight will be undertaken with guidance from the SPLIT ENZ supervisory team. If any further advice is required outside of this team, the PI will approach the ICU research team, where the PI is located.

#### Dissemination Plan

The study protocol is registered with the Australia and New Zealand Clinical Trials registration and is freely available on the web.

At consent, participants will be asked if they would like a copy of the study results, and this will be shared with them after study completion.

Data will be freely shared (with appropriate privacy and confidentiality oversight) at the completion of the study with other health professionals, researchers, and study sponsors as applicable.

## Discussion

### Overview

This study will be the first of its kind in New Zealand and will contribute to understanding of the challenges and level of disability patients face once home, recovering from a critical illness. Despite the appropriate and commendable government response to COVID-19 and the emergence of the Delta, Omicron, and possible future variants, New Zealand traverses a likely future with endemic COVID-19. It is crucial that research is undertaken to understand recovery, survivorship, and quality of life after critical illness. Although this study was originally designed to focus on patients without COVID-19, it is important to recognize that all patients who are critically ill are vulnerable to PICS from a variety of causes and illnesses; hence, both are eligible to be included in this study. Although some authors have postulated both PICS and long COVID-19 are the same entity [73], it is important to highlight that there are some tangible differences among them. For example, there is evidence that many patients infected with SARS-CoV-2 undergo significant acute lung injury leading to chronic dyspnea, with fatigue, mental health and memory problems persisting even in young home-isolated adults who were not even admitted to the ICU [74]. This study will include both patients with COVID-19 and non–COVID-19, with outcomes analyzed separately owing to the differences with long COVID-19 and PICS. Nonetheless, the overall emphasis is on highlighting the recovery journey for New Zealand survivors of critical illness, irrespective of cause of illness.

### Conclusions

SPLIT ENZ is due to begin recruitment at the start of 2022 with an intended recruitment period of no longer than 2 years. The current protocol (version 2.5; November 15, 2021) has undergone an ethics review and Māori consultation and is registered with the Australian New Zealand Clinical Trials Registry.

## Appendix

Multimedia Appendix 1 Peer Review Report from the Health and Disability Ethics Committees, Ministry of Health (New Zealand).

## Abbreviations

COS: core outcome set
GP: general practitioner
ICU: intensive care unit
PerCI: persistently critically ill
PI: principal investigator
PICS: Post Intensive Care Syndrome
PPV: positive pressure ventilation
REDCap: Research Electronic Data Capture
WHODAS: World Health Organization Disability Assessment Scale
