# Correction: Survivorship of Patients After Long Intensive Care Stay With Exploration and Experience in a New Zealand Cohort (SPLIT ENZ): Protocol for a Mixed Methods Study

**DOI:** 10.2196/38180

**Published:** 2022-04-04

**Authors:** Lynsey Sutton, Elliot Bell, Susanna Every-Palmer, Mark Weatherall, Paul Skirrow

**Affiliations:** 1 Intensive Care Unit Level 3, Wellington Regional Hospital Capital and Coast District Health Board Wellington New Zealand; 2 Department of Psychological Medicine University of Otago Wellington New Zealand; 3 Department of Medicine University of Otago Wellington New Zealand

In “Survivorship of Patients After Long Intensive Care Stay With Exploration and Experience in a New Zealand Cohort (SPLIT ENZ): Protocol for a Mixed Methods Study” (JMIR Res Protoc 2022;11(3):e35936) the authors noted one error.

In Textbox 1, one of the exclusion criteria was reported as follows:

- *>18 years*

This has been corrected as follows:

- *<18 years*

The correction will appear in the online version of the paper on the JMIR Publications website on April 4, 2022 together with the publication of this correction notice. Because this was made after submission to PubMed, PubMed Central, and other full-text repositories, the corrected article has also been resubmitted to those repositories.

