# Supplementary material for: Survivorship of Patients After Long Intensive Care Stay With Exploration and Experience in a New Zealand Cohort (SPLIT ENZ): Protocol for a Mixed Methods Study
Source: JMIR Res Protoc. 2022 Mar 17;11(3):e35936. doi: 10.2196/35936 (PMC8972103; doi:10.2196/35936)
Supplement: Multimedia Appendix 1 [file resprot_v11i3e35936_app1.pdf]

Health and Disability Ethics Committees

Health and Disability Ethics Committees
Ministry of Health
C/- MEDSAFE, Level 6, Deloitte House
10 Brandon Street
PO Box 5013
Wellington
6011

0800 4 ETHICS
hdecs@moh.govt.nz

SCIENTIFIC PEER REVIEW:

Date: **20/5/21**

Research Title **SPLIT ENZ: Survivorship post Long Intensive Care stay, Exploration in a New Zealand cohort**

Co-coordinating Investigator: **Lynsey Sutton-Smith**

Peer Reviewer Name: **Dr. Mark Huthwaite**

The protocol was reviewed by *12* academics and their feedback collated in this form. Signed by Dr Mark Huthwaite on behalf of panel of peer reviewers attended who were: Dr. Mathew Jenkins, Francis Goodstadt, Emily Cooney, Zara Mansoor, Gabrielle Jenkin, Renan Lyra, Hannah Paap and the team of supervisors: Dr. Susanna Every Palmer, Dr. Elliot Bell & Dr. Paul Skirrow.

Peer Reviewer Position: Senior Lecturer, University of Otago

Independent from study? Yes

Peer Reviewer signature

Recommendation: Revise minor

**REVIEW GUIDELINE**	**GUIDELINE PROMPTS**	**COMMENTS**	**RESEARCHER RESPONSE**
Relative merit of the research	• Important, worthwhile and justifiable.<br>• Addresses a health issue that is important for health and/or society.<br>• Aims, research questions and hypotheses build on and address gaps in existing knowledge.	Lynsey has clear experience and passion to inform the research. Yes, this is an important topic. Despite an evolving body of international literature there is no current research undertaken in New Zealand, or in Māori patients. The COVID pandemic has highlighted the issue of survivorship and how debilitating Critical Illness recovery can be.	

Design and methods	• Quality of study design<br>• Robustness of the methods used.<br>• Includes a description of sample recruitment and characteristics (including number, gender and ethnicity where relevant) proposed methods of data analysis.<br>• Timelines for the research included	• Robust design which follows other international studies of similar design.<br>• Recommend adjusting the hypothesis to state "For adult ICU patients who have had a prolonged stay in the ICU, a significant proportion will experience moderate to severe disability in the year following critical illness".<br>• Add gender and ethnicity as a covariates.<br>• Consult with Professor Weatherall whether gender matching is necessary to ensure males females are equally represented.<br>• Add a question during the follow up assessments on prior Mental Health issues. This will be important to establish for participants who may not have sought help and/or not have received a formal diagnosis.<br>Consider adding a question around who is receiving ACC funding/home services or not. There may be different outcomes.<br>Outcome measures are appropriate, with WHODAS as primary measure.	Hypothesis has been amended in the protocol.<br>Gender and ethnicity have been added to the covariates.<br>On consultation, Professor Weatherall feels this is unnecessary.<br>Question to be added to the assessment schedule (Dr. Every-Palmer has provided).<br>I will look to see how ACC information can be added, ACC information may only be available by asking participants.
Feasibility of the research	• Overall strategy, methodology and analyses are well reasoned and appropriate to achieve the specific aims of the project.<br>• Likely to improve scientific knowledge, concepts, technical capacity or methods in the research field, or of contributing to better treatments, services, health outcomes or preventive interventions.<br>• Achievable within the specified timeframe<br>• Researcher/research team has the appropriate experience and expertise.	It is an ambitious study but is feasible. **Two point to address:**<br>**1)** Retention could be an issue; high mortality rate in the year following Critical Illness and longitudinal nature of the study may mean large loss to follow up.<br>To have a sufficient sample/data for the primary outcome of interest, you would need 100 participants at 12 months. A suggestion to overcome this would be to oversample during recruitment to ensure 100 patient's data is	Agreed.<br>I will endeavour to oversample during recruitment to achieve 100 participants at 6 month follow up.

		available for analysis at 6 months instead of 12 (whilst continuing to follow participants up at 12 months). This may be more achievable.<br><br>**2)** The qualitative part of the study could be a lot of work with transcripts and analysis. Perhaps the prompts/questions used could be more directive/focussed to reduce the size of the transcripts to the pertinent outcome(s) of interest. It may also be worth considering funding options for transcription.	I will maintain 3 of the "prompt" questions to the pertinent aspects of the research and also look to apply for additional funds for transcription. I am already looking at viable application through a nursing fund scholarship and the New Zealand Nurses Organisation.
Reviewer Independence /objectivity	• Peer review is considered free of bias, equitable and fair.<br>• Objectivity can be compromised if peer reviewers have conflicts of interest, and so appropriate peer reviewers typically will not be materially connected to the researcher(s) in a way that might undermine objectivity, and be free from either positive or negative inducements.<br>• If the peer reviewer is connected to the study please explain what measures are taken to mitigate conflict of interest.	The named peer reviewers consisted of multidisciplinary colleagues with backgrounds in [psychology, psychiatry, health sciences, social sciences). These peer reviewers work in the University of Otago; some have previously met the principal investigator and they know her supervisors. None of the peer reviewers are connected to the study. They were not reimbursed for peer review.	
Other comments	• Any reviewer observations that are not covered in the points above.	• Ensure there is a visual aid the participant can access when the assessments/follow up are happening. This will make it easier to complete and will improve the validity of the self-reporting method. This could be done either by using an app-based approach (Qualtrics) or mail out the paper forms prior to the scheduled follow up. An app may not be appropriate for some patients and those who do not have smart phones/access to a PC (there may be an equity issue using solely this approach).	Email and paper Mail out of key documents will be done 1-2 weeks prior to scheduled follow up. I have developed a cover letter that outlines the key questionnaires and key dates for follow up and this has been added to the protocol.<br>I may look into the use of Qualtrics but acknowledge for my cohort this

		• Consider various methods of follow up – offer face to face interviews for those than can travel and would prefer to.<br>• Utilise family/whānau as support during the interviews.<br>• Ensure participants understand if they lose documents – this will be managed easily with replacements and is not an issue.<br>• Ensure macrons added where appropriate.	option may only suit a small proportion of those with smart phones.<br><br>I will establish participant preference at consent by adding to the consent form.<br>I have added this to the participant/whānau information cover letter that will be emailed/mailed out to them prior to each follow up.
